# Supplementary material for: Complexome profiling of the Chlamydomonas psb28 mutant reveals TEF5 as an early PSII assembly factor
Source: Plant Cell. 2025 Mar 17;37(6):koaf055. doi: 10.1093/plcell/koaf055 (PMC12164590; doi:10.1093/plcell/koaf055)
Supplement: koaf055_Supplementary_Data [file koaf055_supplementary_data.zip › psb28_interactive.zip!/plots/intensity/qualityPlots/peptides/STA1_Cre13.g567950.t1.2.html]

### Cre13.g567950.t1.2

Trivial name: STA1  
SeqCoverage [median]: 11.50  
Mass: 55.61 kDa  
  
 

| Sequence | MissedCleavage | Mass [Da] | wtIntensity | muIntensity | Score |
| --- | --- | --- | --- | --- | --- |
| AKPAVPIGGAYR | 1 | 1198.68 | 1803.71 | 2942.35 | 67.08 |
| ANDFGGEIIPSAAK | 0 | 1388.69 | 406.87 | 2742.00 | 106.49 |
| EAEGIYIR | 0 | 949.49 | 485.82 | 963.30 | 74.42 |
| IVNKEGVTEGTR | 1 | 1301.69 | 1682.37 | 2644.20 | 101.39 |
| SVLLQLLNDSYAK | 0 | 1462.80 | 355.11 | 0.00 | 98.94 |
|  |  |  | 4733.9 | 9291.8 |  |
